# Exploring the Clinical and Genetic Spectrum of Steroid Resistant Nephrotic Syndrome: The PodoNet Registry

**DOI:** 10.3389/fped.2018.00200

**Published:** 2018-07-17

**Authors:** Agnes Trautmann, Beata S. Lipska-Ziętkiewicz, Franz Schaefer

**Affiliations:** ^1^Division of Pediatric Nephrology, University Center for Pediatrics and Adolescent Medicine, Heidelberg, Germany; ^2^Clinical Genetics Unit, Department of Biology and Medical Genetics, Medical University of Gdańsk, Gdańsk, Poland

**Keywords:** SRNS, nephrotic syndrome, NPHS2, WT1, steroid resistant nephrotic syndrome

## Abstract

**Background:** Steroid resistant nephrotic syndrome (SRNS) is a rare condition, accounting for 10–15% of all children with idiopathic nephrotic syndrome. SRNS can be caused by genetic abnormalities or immune system dysfunction. The prognosis of SRNS varies from permanent remission to progression to end-stage kidney disease, and post-transplant recurrence is common.

**Objectives:** The PodoNet registry project aims to explore the demographics and phenotypes of immune-mediated and genetic forms of childhood SRNS, to assess genotype-phenotype correlations, to evaluate clinical management and long-term outcomes, and to search for novel genetic entities and diagnostic and prognostic biomarkers in SRNS.

**Methods:** In 2009, an international registry for SRNS was established to collect retro- and prospective information on renal and extrarenal disease manifestations, histopathological and genetic findings and information on family history, pharmacotherapy responsiveness and long-term outcomes. To date, more than 2,000 patients have been enrolled at 72 pediatric nephrology centers, constituting the largest pediatric SRNS cohort assembled to date.

**Results:** In the course of the project, traditional Sanger sequencing was replaced by NGS-based gene panel screening covering over 30 podocyte-related genes complemented by whole exome sequencing. These approaches allowed to establish genetic diagnoses in 24% of the patients screened, widened the spectrum of genetic disease entities presenting with SRNS phenotype (*COL4A3-5, CLCN5*), and contributed to the discovery of new disease causing genes (*MYOE1, PTPRO*). Forty two percent of patients responded to intensified immunosuppression with complete or partial remission of proteinuria, whereas 58% turned out multi-drug resistant. Medication responsiveness was highly predictive of a favorable long-term outcome, whereas the diagnosis of genetic disease was associated with a high risk to develop end-stage renal disease during childhood. Genetic SRNS forms were generally resistant to immunosuppressive treatment, justifying to avoid such pharmacotherapies altogether once a genetic diagnosis is established. Even symptomatic anti-proteinuric treatment with RAS antagonists seems to be challenging and of limited efficacy in genetic forms of SRNS. The risk of post-transplant disease recurrence was around 30% in non-genetic SRNS whereas it is negligible in genetic cases.

**Conclusion:** In summary, the PodoNet Registry has collected detailed clinical and genetic information in a large SRNS cohort and continues to generate fundamental insights regarding demographic and etiological disease aspects, genotype-phenotype associations, the efficacy of therapeutic strategies, and long-term patient and renal outcomes including post-transplant disease recurrence.

## Introducing the podonet registry study for steroid resistant nephrotic syndrome

While most children with idiopathic nephrotic syndrome respond to oral glucocorticoid therapy and have a favorable long-term prognosis, ~10–15% of children do not respond and are classified as steroid resistant nephrotic syndrome (SRNS). SRNS is a rare (incidence 3–4 per million person-years) and challenging clinical condition with heterogeneous etiology and highly variable disease courses and outcomes.

Whereas, a relevant fraction of SRNS children respond to intensified immunosuppressive therapy with temporary or persistent proteinuria remission, others exhibit primary multidrug resistance. Traditionally, the diagnostic and prognostic assessment of SRNS has been based on histopathological categorization. In recent years however, the understanding of SRNS pathophysiology has transformed fundamentally with the discovery of a rapidly growing number of genetic disorders of the podocytes. These novel insights have created a need to redefine diagnostic assessment, prognostic classification and therapeutic approaches in childhood-onset SRNS. However, clinical research in SRNS is compromised by the low incidence and heterogeneity of the disorder. To overcome the limitations related to the rarity of the disorder, the PodoNet Consortium set up an international web-based registry for childhood SRNS and CNS (www.podonet.org).

The objectives of the PodoNet registry project are to explore the demographics and phenotypes of immune-mediated and genetic forms of childhood SRNS, to evaluate genotype-phenotype correlations, to evaluate clinical management and long-term outcomes, and to search for genetic entities and novel diagnostic and prognostic biomarkers of SRNS. Data collection is focused on the characteristics at first disease manifestation, genetic and histopathological findings, the responsiveness to pharmacological therapies, long-term renal survival, and post-transplant disease recurrence.

This manuscript reviews the accomplishments of the international PodoNet project since 2009, focusing on both genetic and clinical aspects of SRNS. The international PodoNet registry study with retro- and prospective data of currently more than 2,000 patients provides extended knowledge about SRNS demographics, SRNS genotype-phenotype correlations, therapeutic and prognostic outcomes and has discovered new genetic disorders. These results contribute to an improved understanding of the different SRNS entities and to a re-classification of SRNS based on molecular etiology and responsiveness to second-line treatment.

## Podonet study cohort: methods and definitions

**Figure 1:**
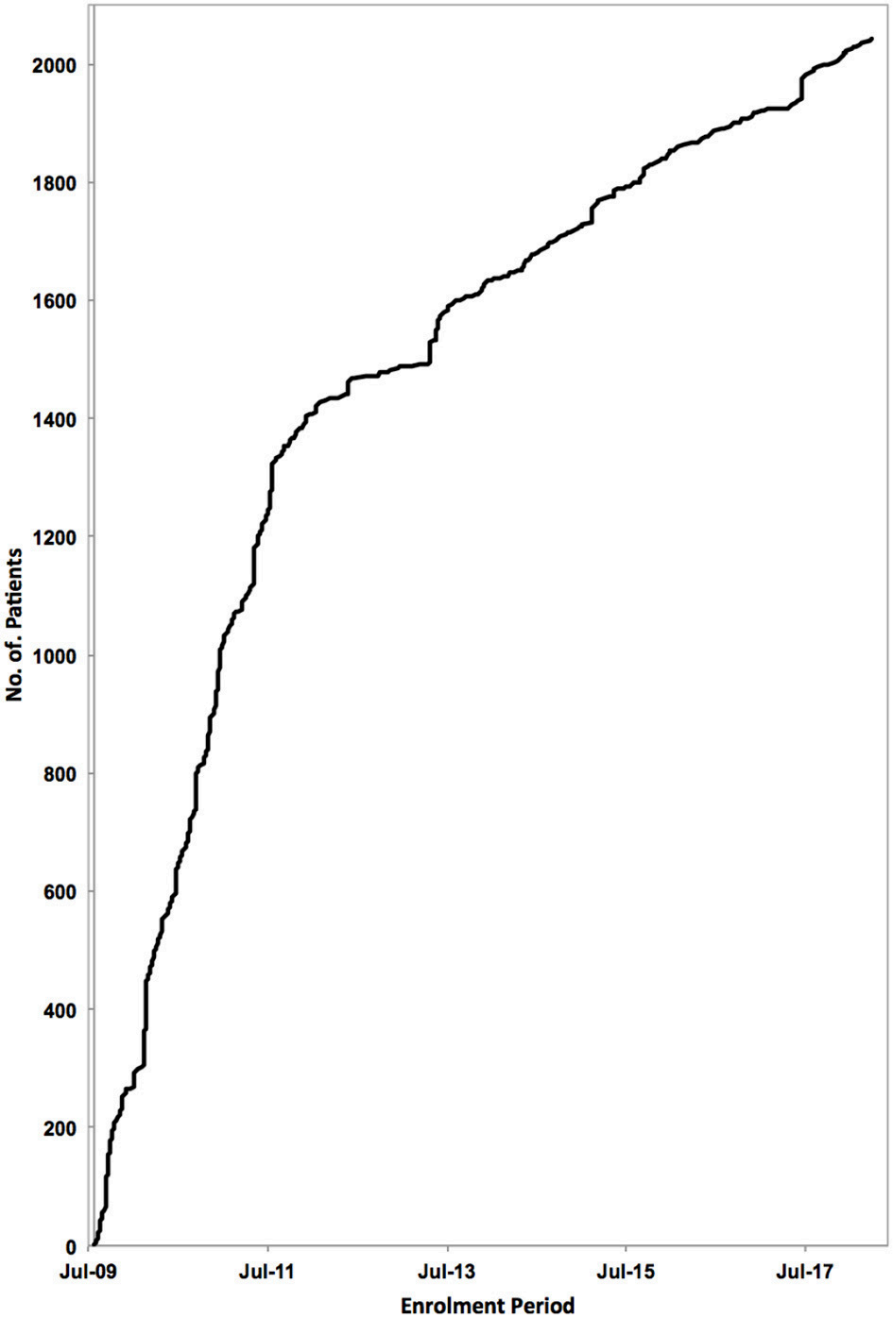
Patient enrolment to the PodoNet Registry. 2,041 children with SRNS have been registered at 72 clinical pediatric nephrology centers in 28 countries since August 2009.

**Figure 2:**
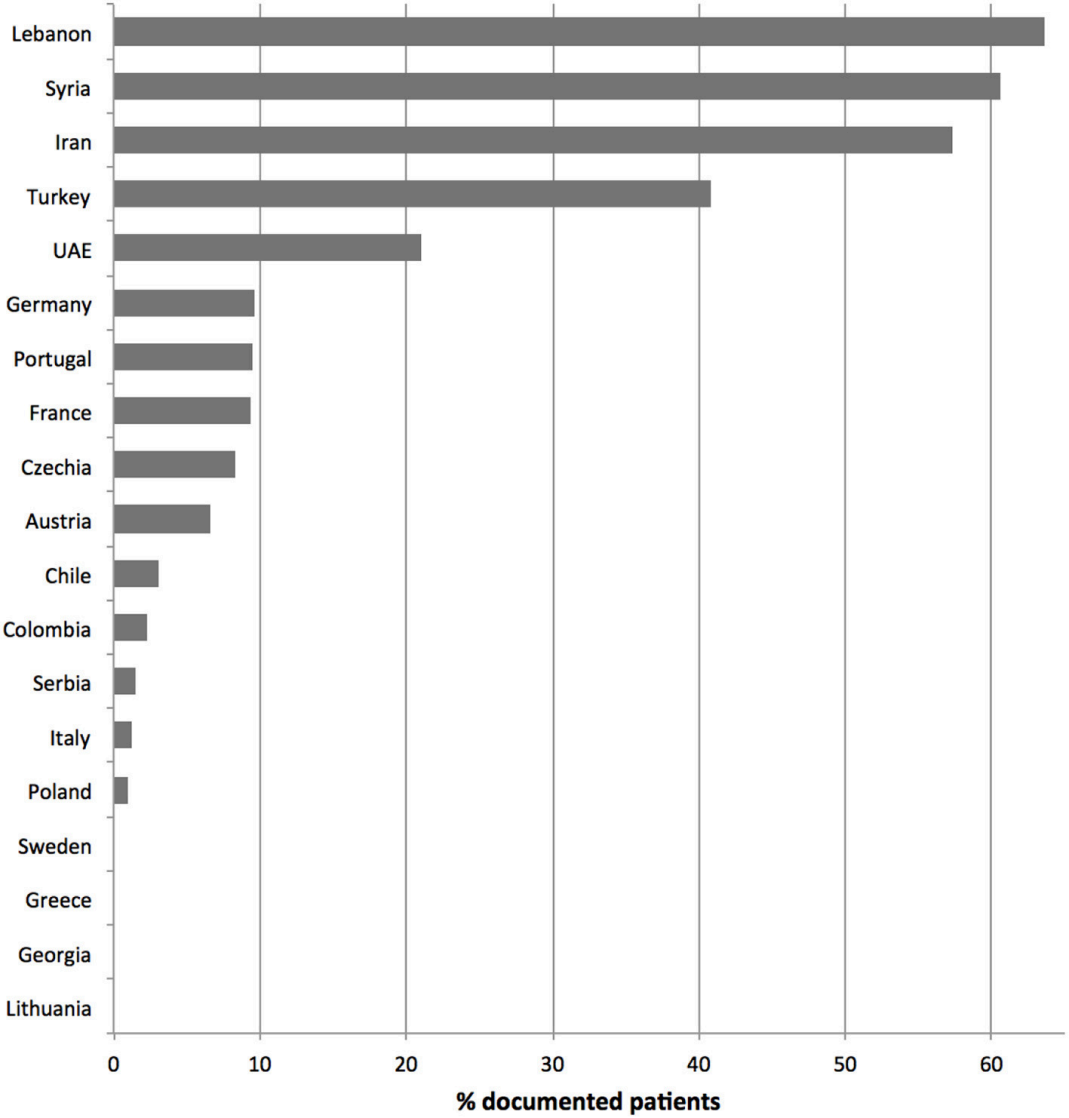
Documented parental consanguinity per country. Only countries with more than 10 registered patients were considered.

The PodoNet registry is a web-based clinical database (www.podonet.org) for primary steroid resistant (SRNS) and congenital nephrotic syndrome (CNS). The registry follows patients with childhood-onset (age < 20 years) primary SRNS, CNS or persistent subnephrotic proteinuria with likely genetic disease, but not patients with secondary SRNS. Since August 2009, investigators from 72 clinical units in 28 countries have enrolled 2041 patients (Figure 1). The patients derive mainly from European countries (~85%) but also from the Middle East and Latin America (15%) (1). Consanguinity and familial disease occurrence were most common in the Middle Eastern countries (Figure 2). Details on the structure of the PodoNet study cohort along with the registry study protocol and description were recently published (1).

Genetic screening was initially performed using Sanger sequencing of individual genes following a diagnostic screening algorithm based on age at presentation and extrarenal symptoms. From 2013 onward, next generation panel sequencing (NGS) of more than 30 podocytopathy-associated genes was systematically applied. Details of the diagnostic genetic screening studies and their completeness are described below.

Intensified immunosuppressive (IIS) therapies applied after confirmation of steroid resistance (persistent nephrotic-range proteinuria after 4 weeks oral prednisone at 60 mg/m^2^ per day), included intravenous steroid pulses, calcineurin inhibitors (CNI), mycophenolate-mofetil (MMF), the combination of CNI and MMF, oral and intravenous cyclophosphamide (CPH), and rituximab.

The response to IIS (complete, partial, no remission) was assessed using a standardized set of criteria using changes in proteinuria and serum albumin, as previously defined (1, 2). To categorize IIS responsiveness as a basis for predicting long-term outcomes, the evaluation period was restricted to the first year of IIS treatment and disease. Patients with CNS were excluded. If patients were treated with more than one intensified immunosuppressive treatment in the first year, the most efficacious treatment and the best antiproteinuric response was identified. Patients who were unresponsive to any IIS applied were classified as multi-drug resistant.

End-stage kidney disease (ESKD) was defined by attainment of CKD stage 5 and/or start of renal replacement therapy.

All statistical analyses of the reported individual studies of the PodoNet registry were performed using SAS® Version 9.4 (Cary, USA). Statistical details can be obtained from the original articles (1–5).

## Clinical and genetic spectrum of the podonet cohort study—results and discussion

### Clinical aspects of SRNS

#### First manifestation

**Figure 3:**
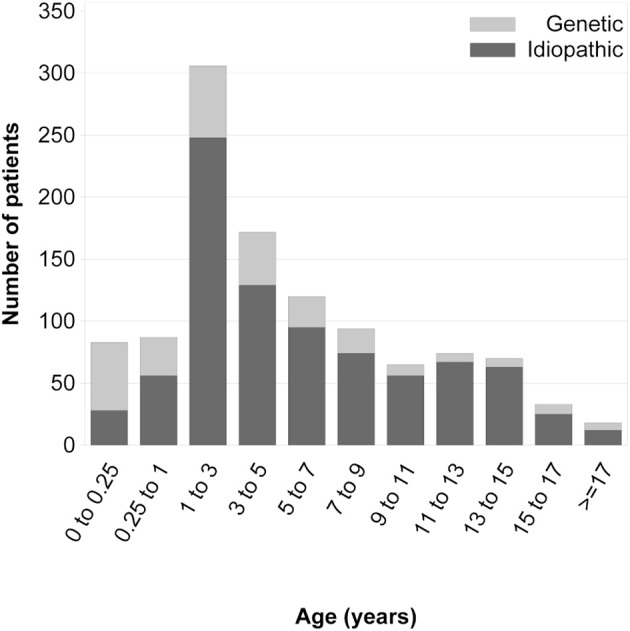
Age at first disease manifestation in children with and without an identified genetic cause of steroid-resistant nephrotic syndrome [with copyright permission (1)].

Among all patients reported to the PodoNet Registry, 6% presented with congenital nephrotic syndrome, 7% manifested as early infantile nephrotic syndrome (onset age 3–11 months), 51% at 1–5 years, 23% at 6–11 years, and 13% at age 12 years and older (Figure 3).

Hypoalbuminemia was most pronounced in congenital nephrotic syndrome (mean serum albumin 17 g/L) and least marked in adolescent-onset disease (26 g/L). Likewise, hypertension at diagnosis was most prevalent in adolescents (28%) as compared to 14% in infants. A small fraction of patients (8.7%) were diagnosed with non-nephrotic proteinuria, but progressed to full nephrotic syndrome during follow-up (1).

63.5% of children had a normal renal function (CKD stage 1), 23.5% a mildly impaired renal function at first presentation. Chronic kidney disease (CKD stage 3 and 4) was present in 13% (1). 7.4% of patients progressed to ESKD in the first year, whereas the median time to ESKD was 2.8 years.

Extrarenal disease manifestations pointing to a syndromic disorder most commonly included neurological symptoms (5.3% including brain anomalies, microcephaly and/or mental retardation), short stature (5.1%) and facial dysmorphism (2.2%).

#### Histopathological findings

**Figure 4:**
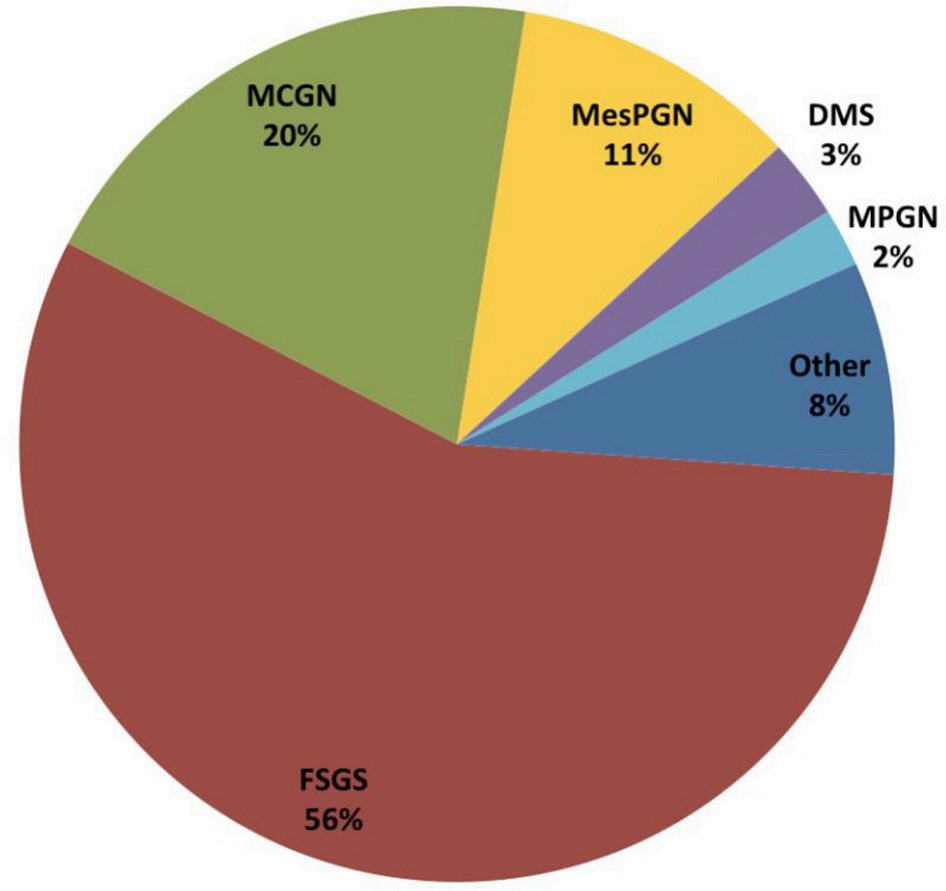
Distribution of underlying histopathologic diagnosis in the first renal biopsy performed in 1,651 children with SRNS from the PodoNet cohort.

The predominant histopathological diagnosis was FSGS in 56% of all PodoNet patients, followed by minimal-change nephropathy (MCN) with 20% and mesangioproliferative GN (MesPGN) with 11% (Figure 4). The predominance of FSGS was also found in other SRNS populations (6–9), albeit with some variability likely related to variations of ethnic composition, biopsy indication policies, and disease duration at time of biopsy.

Twelve percent of the PodoNet patients received a second renal biopsy during the course of the disease. Two thirds of the re-biopsied patients previously diagnosed with MCN or MesGN showed FSGS in the second biopsy, and 10% of those with FSGS progressed to global glomerulosclerosis (GGS).

The children in our cohort who initially presented with FSGS, MCN, or MesPGN displayed similar severity of hypoalbuminemia (25–27 g/dl) and comparable prevalence of hypertension (15–18%). Patients with FSGS were on average slightly older (6 years vs. 4 years for MCN/MesPGN) and presented with a slightly lower eGFR than patients with MCN/MesPGN.

The other histopathological entities found in children with SRNS - diffuse mesangial sclerosis (DMS, 3%), membranoproliferative glomerulopathy (MPGN, 2%), membranous nephropathy (MN, 2%)—were much less common. Typically, patients with these diagnoses manifested with milder initial hypoalbuminemia but slightly higher prevalence of hypertension (23–26%) than patients with FSGS, MCN and MesPGN. Patients with DMS usually presented at earlier age (median age of 1.3 years), similar to the observations of the SRNS Study Group where DMS was found in 27% of infants (10) with established renal failure and an identified genetic cause in almost two thirds of cases (1). In patients with FSGS, the detection rate of genetic disease was lower with 22% and lowest with 12% in children with MCN (1).

### Genetic aspects of SRNS

#### Diagnostic screening studies

To date, a total of 1,554 individual PodoNet patients were genetically screened for hereditary podocytopathies. Initially, the most commonly screened genes were *NPHS2* and *WT1* performed by Sanger sequencing whereas other podocyte genes were screened more selectively guided by age, histopathology, and/or syndromic features. These included *WT1* disease (sex reversal/urogenital abnormalities and malignancies), mitochondrial dysfunction (myopathy, cardiomyopathy, impaired hearing), Pierson syndrome (impaired vision), and Schimke syndrome (osteodysplasia) (1).

Since 2014, next generation sequencing (NGS) gene panels were used to systematically screen for all known podocytopathy genes as part of the EURenOmics project (www.eurenomics.org). Identified mutations in the panel sequencing were confirmed by Sanger sequencing. To date, 539 unrelated patients with documented multi-drug resistance or unknown responsiveness to intensified immunosuppressive therapy were screened by NGS panel including more than 30 podocyte specific genes. These included 214 novel consecutively enrolled cases and 315 previously tested negative by conventional screening of *NPHS2* and exons 8–9 of *WT1*.

Overall, since the beginning of the PodoNet Registry genetic diagnoses were established in 373 SRNS patients (24% of those tested; 19% of the entire cohort). This mutation detection rate is slightly lower compared to that recently reported in a study of 1783 SRNS families (29.5%) (10).

In line with other SRNS studies (9, 10), mutations in *NPHS2, WT1* and *NPHS1* represented the most common genetic SRNS causes, accounting for 42, 16, and 13% of cases, respectively (Table 1). Using the NGS gene panel, mutations were identified in 27% of novel consecutive patients and in 17% of patients previously found negative for *NPHS2* and exons 8–9 of *WT1* mutations. Hence, almost half of all genetic SRNS patients carry a mutation in *NPHS2* or the zinc finger domains of *WT1*, which could provide a rationale for a cost-effective two-step mutational screening algorithm.

**Table 1:** Mutation screening results.

**Causative gene**	***N***	**%**
*NPHS2*	156	41.8
*WT1*	59	15.8
*NPHS1*	47	12.6
*SMARCAL1*	20	5.4
*PLCE1*	14	3.8
*ADCK4*	13	3.5
*COL4A5*	11	1.3
*LMX1B*	10	2.7
*INF2*	8	2.1
*LAMB2*	8	2.1
*COQ6*	6	1.6
*MYO1E*	6	1.6
*PTPRO*	5	1.3
*TRPC6*	3	0.8
*CLCN5*	2	0.5
*COQ2*	2	0.5
*PAX2*	2	0.5
*ACTN4*	1	0.3
*COL4A3*	1	0.3
Others	4	1.1
All	373	100

*Genetic diagnoses in 373 of 1,554 genetically screened children with hereditary SRNS (24%)*.

The mutation detection rate was highest in children with CNS and significantly lower with increasing age at first manifestation during the first 6 years of life (Figure 3), in keeping with observations by the SRNS Study Group (10) and the German Pediatric Nephrology Society (9). While genetic screening strategies for diagnosing childhood-onset nephrotic syndrome were generally established when the PodoNet registry was founded, screening strategies for adolescent-onset disease were not well-established. Utilizing our PodoNet cohort, 297 patients with non-syndromic SRNS and disease onset in the second decade of life were screened for mutations in *NPHS2, WT1, TRPC6, ACTN4*, and *INF2* (3). Seventy-nine percent of adolescents had a sporadic disease occurrence, 21% familial disease occurrence (17% autosomal-recessive, 4% autosomal-dominant forms). The overall mutation rate in the adolescent age group in those five genes was 11%, separated into 30% in the autosomal-dominant cases, 13% in the autosomal-recessive and 10% of the sporadic cases. *NPHS2* mutations accounted for 7% of all adolescent cases. Based on the common involvement of p.R229Q in late-onset SRNS, an approach of a two-step screening algorithm (limiting full sequencing of the *NPHS2* gene to carriers of the p.R229Q polymorphism) was suggested in previous studies (11, 12). As only 56% of adolescents with *NPHS2*-associated disease were compound-heterozygous for a mutation combined with the p.R229Q polymorphism in the PodoNet cohort, we suggested to rather screen the entire coding sequence of *NPHS2* in all sporadic and autosomal-recessive cases of juvenile SRNS. The considerations of selective and limited genetic screening in adolescents subsequently became largely obsolete with the advent of next generation sequencing.

#### Gene exploration studies

One of the aims of the PodoNet Network is to foster the identification of new SRNS causing genes. Registered PodoNet patients were evaluated regarding familial disease occurrence and negative screening in the known SRNS genes and biomaterial was collected. This allows to contribute and to perform whole-genome linkage analysis and high-throughput sequencing in affected families. PodoNet Consortium partners have identified two new SRNS causing genes participation of SRNS families of the PodoNet cohort in 2011: *PTPRO* (13) and *MYO1E* (14).

##### *PTPRO*-associated SRNS

The role of the protein tyrosine phosphatase receptor type O (*PTPRO*) gene, also known as *GLEPP1* (glomerular epithelial protein 1) gene, is the regulation of glomerular pressure and permselectivity. The protein PTPRO is a tyrosine phosphatase expressed at the apical membrane of the podocyte foot processes. Thyrosine phosphorylation of tight junction proteins plays a major role in controlling paracellular permeability, cell signaling and actin cytoskeleton remodeling.

*PTPRO* mutations were identified in 5 of 29 affected children from two of 17 tested families with so far unknown genetic disease. The disease onset varied between 5 and 14 years, the initial serum albumin varied between 19 and 40 g/L at first manifestation. Apart from one affected child, none of the children had progressed to ESKD 2–5 years after first manifestation. Renal biopsy, performed in 2 children, revealed MCN as well as FSGS (13) and electron microscopy showed diffuse podocyte foot process fusion and extensive microvillus transformation of podocytes. The subsequent testing of almost 2000 SRNS cases of all age groups performed within the frames of the EuRenOmics project did not identify any further individuals with pathogenic biallelic *PTPRO* mutations, demonstrating its extremely rare character.

##### *MYO1E*-associated SRNS

*MYO1E* encodes a non-muscle class I myosin (Myo1E) which is expressed mainly at the podocyte plasma membrane in the glomerulus. The role of Myo1E is maintaining the function of the glomerular filtration barrier and promoting the podocyte motility. Two mutations in *MYO1E* were identified in two families (3 siblings, 1 other child), which are closely associated with autosomal recessive SRNS/FSGS (14).

The age at disease onset of the index patient was 9 years; the siblings and the fourth child were diagnosed earlier (between age of 1–4 years). The histopathological diagnosis was FSGS in all children, electron microscopy showed thickening and disorganization of the glomerular basement membrane.

All four patients showed generally non-responsiveness to intensified immunosuppression. Cyclosporine A therapy was associated with a transient proteinuria reduction in 2 of 4 children, possibly related to a direct podocyte cytoskeleton stabilizing effect of calcineurin inhibition. One affected member progressed to ESKD at age of 13 years; the other affected siblings had milder disease courses, possibly related to earlier diagnosis and/or to effective antiproteinuric treatment. Three further unrelated individuals with *MYO1E* biallelic mutations were subsequently identified through NGS gene panel screening, all of them presented in early infancy.

### Genotype-phenotype association studies

#### WT1-associated SRNS

*WT1* mutations are associated with a wide range of clinical phenotypes. The PodoNet consortium explored the phenotypic spectrum and analyzed potential genotype-phenotype correlations in 61 patients with *WT1*-associated SRNS, the largest cohort studied to date (4). Both renal and extrarenal phenotypes were found to be clearly associated with the type and location of the causative *WT1* mutation.

55 of 61 patients (90%) carried mutations in the *hot spot region* (exons 8 and 9 and their intronic junctions). The mutations were categorized as exonic mutations (40/61 patients) including truncating mutations, DNA-binding-site mutations and other missense mutation and as intronic mutations (21/61 patients, KTS intron 9 and other intronic mutations). The two largest subgroups (70%) are exonic mutations affecting the nucleotides coding for DNA-binding residues and intronic (9) KTS mutations, the latter are classically associated with Denys-Drash- and Frasier-syndrome.

Patients with exonic mutations were significantly younger at diagnosis (1.1 vs. 4.5 years), presented with more severe proteinuria, edema, and hypertension and progressed more rapidly to ESKD (5-year renal survival from diagnosis 36 vs. 85%) and had a higher risk for nephroblastoma (73 vs. 19%) than patients with intronic mutations. Gonadoblastoma occurs less frequently than Wilms tumors and are more likely to develop in patients with sex reversal commonly associated with intronic mutation.

Missense mutations affecting the DNA-binding site were associated with diffuse mesangial sclerosis (74%), early steroid-resistant nephrotic syndrome onset [0.9 (0.2–1.6) years] and rapid progression to ESKD. Truncating mutations implicated the highest Wilms tumor risk (78%) but had typically late-onset SRNS [12.3 (0.6–15.3) years]. Intronic (KTS) mutations were most likely to present as isolated SRNS (37%) with a median onset at the age of 4.5 (3.1–8.1) years, FSGS (67%) and slow progression (median ESKD age 13.6 years). All patients with isolated SRNS were genotypic and phenotypic females.

In contrast to the consistent associations of mutation type and clinical disease manifestation, histopathology showed high diagnostic and prognostic variability. Furthermore, genital and urinary tract abnormalities varied widely, from hypospadias and unilateral cryptorchidism to global penoscrotal hypoplasia. Genital malformations were detected in 50% of all patients. Male-to female sex reversal occurred exclusively in patients with intronic KTS mutation and exonic DNA-binding site mutation.

We concluded that establishing the genetic diagnosis and identifying the mutation type and localization is important to assess the associated features and complications. The important impact for the clinical decision is the consideration when is the appropriate timing to perform bilateral nephrectomy or gonadectomy to prevent the development of malignancies.

#### SMARCAL1-associated SRNS

Schimke immuno-osseous dysplasia (SIOD), caused by mutations in *SMARCAL1*, is a rare multisystem disorder characterized by the combination of progressive proteinuric glomerulopathy with spondyloepiphyseal dysplasia, growth retardation, dysmorphic features, episodic neutro-/lymphopenia and thrombocytopenia, defective T-cellular immunity, autoimmune disorders, abnormal skin pigmentation, and cerebral infarcts. Early mortality due to severe opportunistic infections or cerebral events is common.

The PodoNet consortium analyzed the renal and extrarenal phenotypic spectrum and genotype-phenotype associations in 34 PodoNet patients from 28 families—the largest *SMARCAL1*-associated nephropathy cohort to date (5). The diagnosis of *SMARCAL1*-associated disease was made either by the presence of typical syndromic feature or unexpectedly by NGS panel screening (11/34 children). All patients diagnosed incidentally through NGS screening were short in stature (height SDS −3.2 ± 1.5) but these patients developed a milder if any extrarenal phenotype during follow-up, with 88% 10-year patient survival as compared to 40% in the patients diagnosed by typical SIOD features.

The timing of proteinuria onset (median age 4.5 (IQR 3.2–7.2) years, nephrotic-range in 69% of children) and the rate of progression to ESKD were similar in all patients. Median age at ESKD was 8.7 (IQR 5.6–10) years. Renal biopsy showed FSGS in 81.5% and MCN in the remaining patients. The extrarenal symptoms, but not the renal phenotype, correlated with the type of *SMARCAL1* genetic mutation.

Since the study demonstrated that in a substantial number of patients SIOD initially presents as isolated SRNS with short stature, it was concluded that *SMARCAL1* screening should be performed routinely also in non-syndromic SRNS and should be included into the SRNS gene panels.

#### ADCK4-associated SRNS

Mutations in *ADCK4* (also known as *COQ8*) were recently identified as a novel autosomal-recessive cause of adolescent-onset SRNS caused by defective Coenzyme Q_10_ biosynthesis in podocytes. Hereditary defects of CoQ_10_ biosynthesis can cause SRNS as part of multiorgan involvement or as isolated SRNS.

Systematic *ADCK4* screening was performed in large cohort of 534 SRNS patients, including 202 patients of the PodoNet cohort (15). *ADCK4* mutations were identified in 26 patients from 12 families with a mutation detection rate of 1.9%. The disease almost exclusively manifested during adolescence, typically with subnephrotic to nephrotic-range proteinuria with no or mild edema but with advanced CKD in 46% of patients progressing to ESKD within a median of 9 months after diagnosis. Renal biopsies revealed FSGS in all biopsied patients. *ADCK4* mutations showed a mainly renal-limited phenotype, with mild neurological features in 5 of 26 patients (occasional seizures, mild mental retardation, retinitis pigmentosa).

*ADCK4*-associated glomerulopathy is the first hereditary form of SRNS with a potentially causative molecular therapy. Oral supplementation of CoQ_10_ reduces proteinuria and stabilizes renal function when applied early in the course of disease (16). Therefore, *ADCK4* mutation screening should be included into NGS screening of all patients with adolescent-onset proteinuric kidney disease.

#### COL4A3-5-associated SRNS

There is increasing evidence for overlapping phenotypes caused by abnormalities in the *COL4* genes and those primarily associated with SRNS/FSGS (17–20).

Among 481 patients of the PodoNet Cohort screened in *COL4A3-5*, 11 carried pathogenic mutations in *COL4A5*, and one was homozygous for a mutation in *COL4A3*.

The detection rate of 2.5% is identical to a recent pediatric study from the UK, where disease-causing mutations in *COL4A3-5* genes were identified in six out of 255 individuals with an SRNS phenotype (19). Other studies found even higher rates of *COL4* mutations particularly in adult SRNS cohorts, but these included heterozygous variants in *COL4A3/4* which are unlikely to cause severe and progressive proteinuric kidney disease (17, 18). In line with other recent studies, all patients with *COL4* gene mutations in our cohort showed FSGS on biopsy and no extrarenal disease manifestations were reported (17–19). These findings support the notion that genetic abnormalities collagen type IV formation may result in a phenotype of isolated nephrotic range proteinuria.

#### CLCN5-associated nephropathy

A novel mutation in the *CLCN5* gene (c.2000delC) was detected by exome sequencing in a family from the PodoNet cohort with apparently isolated nephrotic range proteinuria that had been classified as familial SRNS on clinical grounds. Both affected children were screened negative in 31 glomerulopathy-related genes. Mutations in *CLCN5*, which encodes a voltage-gated chloride ion channel expressed selectively in the renal tubule, are the major cause of Dent disease. Dent disease is an X-linked proximal renal tubular disorder; the key features include low-molecular weight proteinuria, hypercalciuria, nephrocalcinosis, and/or nephrolithiasis.

The genetic finding triggered clinical investigation for a putative tubular defect, which revealed asymptomatic hypercalciuria in only one of two children studied. In the absence of nephrocalcinosis and nephrolithiasis, Dent disease was not considered as a potential differential diagnosis of nephrotic-range proteinuria before. The genetic classification of the disease has important therapeutic and prognostic implications for the affected children. The children had been (mis)-classified as familial SRNS based on the observed nephrotic range proteinuria (>1 g/m^2^ per day). Our case illustrates the genetic and phenotypic heterogeneity of proteinuric kidney diseases. The remarkable phenotypic variability of mutations in *CLCN5* suggests that this gene should be included in NGS panel screening for hereditary proteinuric disorders.

## Treatment and long-term outcome of SRNS

### Responsiveness of SRNS patients to intensified immunosuppression

**Figure 5:**
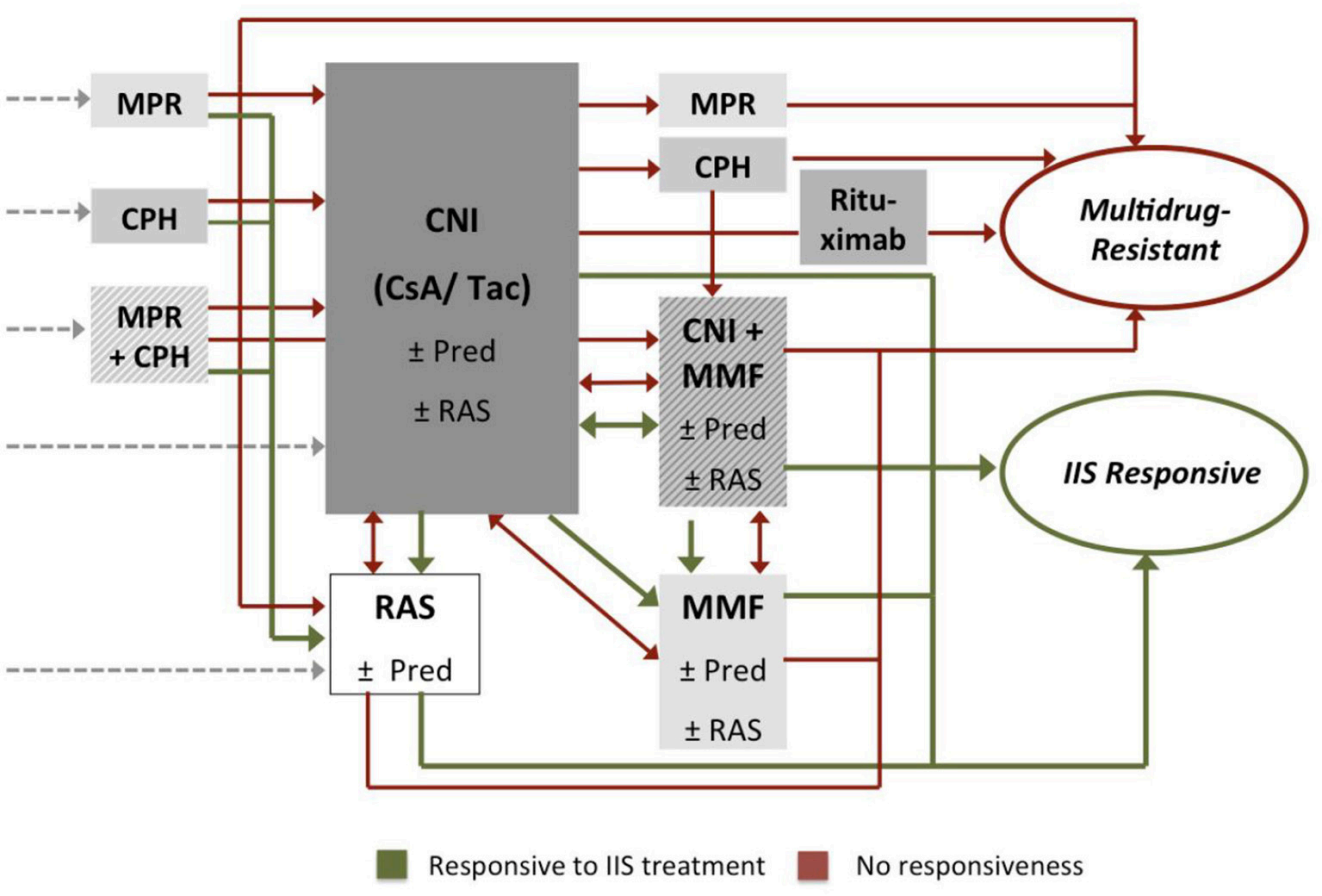
Immunosuppressive treatments applied following diagnosis of primary steroid resistance: oral prednisolone (Pred), methylprednisolone pulses (MPR), calcineurin inhibitors (CNI), mycophenolate-mofetil (MMF), cyclophosphamide (CPH). Approximately 50% of children were co-treated with renin-angiotensin-aldosterone-system inhibitors (RAS). Patients responding to the intensified immunosuppressive (IIS) treatment were classified as IIS responsive (green), others as multi-drug resistant (red).

Whereas, the initial treatment of idiopathic nephrotic syndrome with oral glucocorticoids is well-established, we observed a large variety of the intensified immunosuppressive protocols and algorithms applied following the diagnosis of primary steroid resistance (Figure 5). In an analysis of immunosuppressive therapies applied in 612 SRNS patients in the first year after diagnosis of steroid resistance (2), 62% of children were treated with a single treatment protocol, 28% with two, and 10% with three or more different immunosuppressive drug combinations as a polypragmatic therapeutic approach. While approximately three quarters of patients received Calcineurin inhibitors as first line therapy, others were administered intravenous steroid pulses, mycophenolate mofetil, oral or intravenous cyclophoshamide, or Rituximab (Figure 5). More than 80% of patients were co-treated with oral steroids initially, and more than 70% with renin angiotensin aldosterone system (RAS) antagonists.

**Figure 6:**
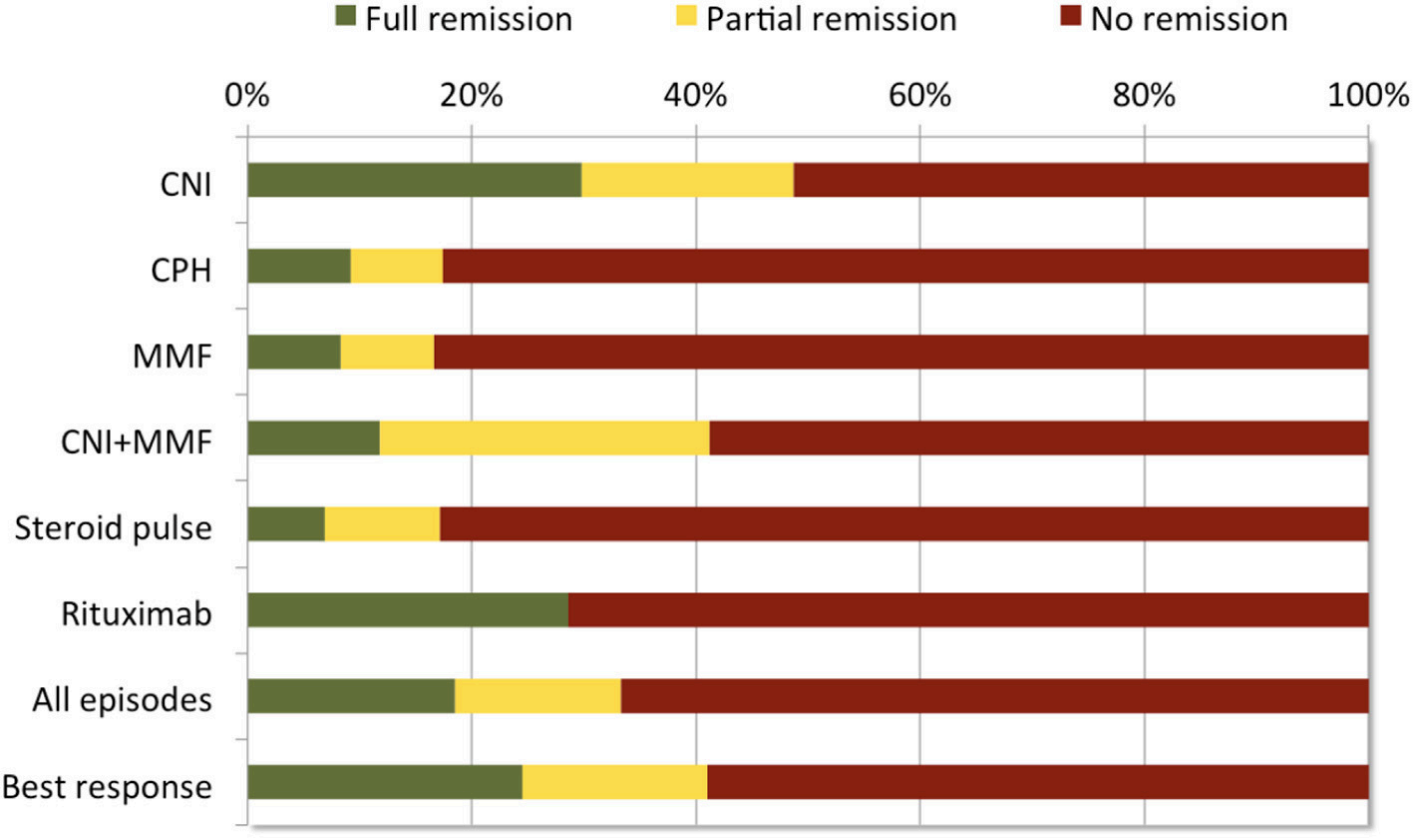
Response to IIS treatment episodes during first year after disease onset in 612 children with SRNS. In total, 232 (38%) patients were treated with more than one treatment protocol during the first year after disease onset. Most efficacious treatment (=best response) was used to classify patients [adapted from Table 2, (2)].

Applying a standardized set of criteria to define treatment response and remission, we observed only 41% of included 612 SRNS patients to respond to any intensified immunosuppression with a relevant proteinuria reduction to IIS (24% with complete, 17% with partial remission), whereas 59% of patients remained unresponsive—often to several therapeutic interventions (Figure 6). The highest rates of complete (30%) or partial (19%) remission were achieved with CNI based protocols. These response rates are in the lower range of previously reported treatment experience, where complete remission was observed in 31% to 89% and partial remission in 19 to 38% of patients (9, 21–25). The variation of the response rates may be related to the selection and composition of the individual study cohorts and the chosen set of response criteria.

The efficacy of CNI-based therapies in the PodoNet cohort was by far superior to steroid pulses, cyclophosphamide, and MMF monotherapy, all of which did not show any therapeutic effect in around 85% of patients as first line therapy (Figure 6) and were completely non-efficacious as second- or third-line therapies in CNI resistant patients. These findings confirm previous studies (26–31) and provide strong evidence against the use of these therapeutics in SRNS. Notably, B-cell depleting therapy with Rituximab induced complete remission in 44%, and partial remission in 15% of patients (1).

RAS inhibition has been demonstrated to lower proteinuria by 40–50% in patients with SRNS (32, 33). In the PodoNet cohort, RAS inhibition alone was associated with partial proteinuria remission in 21% and even maintained complete remission in 27% of patients (1). Hence, the frequent co-administration of RAS antagonists with immunosuppressive agents is a major confounder to the efficacy analysis of the latter.

Among the 602 cohort patients available for pharmacotherapy analysis 82% had sporadic disease, 74 (12%) confirmed genetic disease and 36 (6%) familial disease with negative genetic screening in all known podocytopathy genes.

### Do patients with genetic SRNS respond to immunosuppression?

Anecdotal clinical observations in previous case series and small retrospective studies suggested that individual patients with pathogenic mutations in podocytopathy genes might show some responsiveness to intensified immunosuppression. In total, 11 hereditary SRNS patients reported in literature developed complete remission and 17 partial remission of proteinuria while on calcineurin inhibitor and usually concomitant RAS antagonist therapy (4, 34–38). A non-immunological antiproteinuric action of Ciclosporin A (CsA) mediated by stabilization of the actin cytoskeleton has been suggested based on experimental findings (39).

The PodoNet registry allowed to evaluate the largest cohort of children with hereditary podocytopathies to date exposed to calcineurin inhibitor and other immunosuppressive therapy. Complete or partial proteinuria responsiveness to pharmacotherapy was observed in 45% of children with sporadic disease and in 47% of those with familial, gene screening negative disease, whereas only 13% of patients with hereditary podocytopathies displayed any, mostly transient responsiveness to pharmacotherapy (2).

Transient complete remission was documented in only two out of 74 patients (2.7%) diagnosed with genetic disease in whom extended treatment and response data were available: one child with a *WT1* mutation reportedly achieved complete remission for 2 weeks after start of CsA, followed by subnephrotic range proteinuria for > 11 years. Another child with *NPHS2*-associated disease achieved complete remission for 4–6 weeks, followed by a relapse and subsequently persistent proteinuria with progression to ESKD within 4 years. Transient partial remission with reduction of proteinuria to the non-nephrotic range was observed in response to CsA in another 8 patients (10.8%) with genetic disease (4 *NPHS2*, 3 *WT1*, 1 *COQ6*). Five of these returned to nephrotic-range proteinuria within <2.5 years, and four progressed to CKD stage 3–5 within <5 years. Importantly, 4 of 8 patients were co-treated with RAS antagonists. It is impossible to differentiate whether the transient responsiveness was related to calcineurin inhibition, supportive antiproteinuric RAS antagonist therapy or the natural course of disease with diminishing proteinuria due to worsening renal function. Notably, almost all patients with genetic disease and apparent CsA responsiveness progressed to ESKD soon despite ongoing therapy.

Hence, the findings in the PodoNet cohort argue against a relevant nephroprotective effect of calcineurin inhibition—or other immunosuppressive therapies—in children with genetic forms of SRNS and support the notion that such patients should be spared immunosuppressant side effects. On the other hand, the knowledge about the heterogeneity of underlying genetic disease mechanisms might help to develop specific podocyte-protecting treatment strategies in order to minimize proteinuria. A promising example of an innovative gene specific treatment option is successful use of CoQ10 in children with SRNS due to genetic defects leading to CoQ10 deficiency (15, 16, 40). In the light of these findings and given the progress in the speed, comprehensiveness and cost efficacy brought about by next-generation sequencing, genetic screening should be considered in all SRNS patients at the time of diagnosis of steroid resistance prior to starting intensified immunosuppressive treatment.

Finally, the efficacy of other supportive and/or preventive treatment strategies including antiproteinuric therapies and treatment of hyperlipidemia have not yet been studied in detail in the PodoNet or other cohorts and await exploration in future studies.

### Long-term outcome and risk factors for ESKD

**Figure 7:**
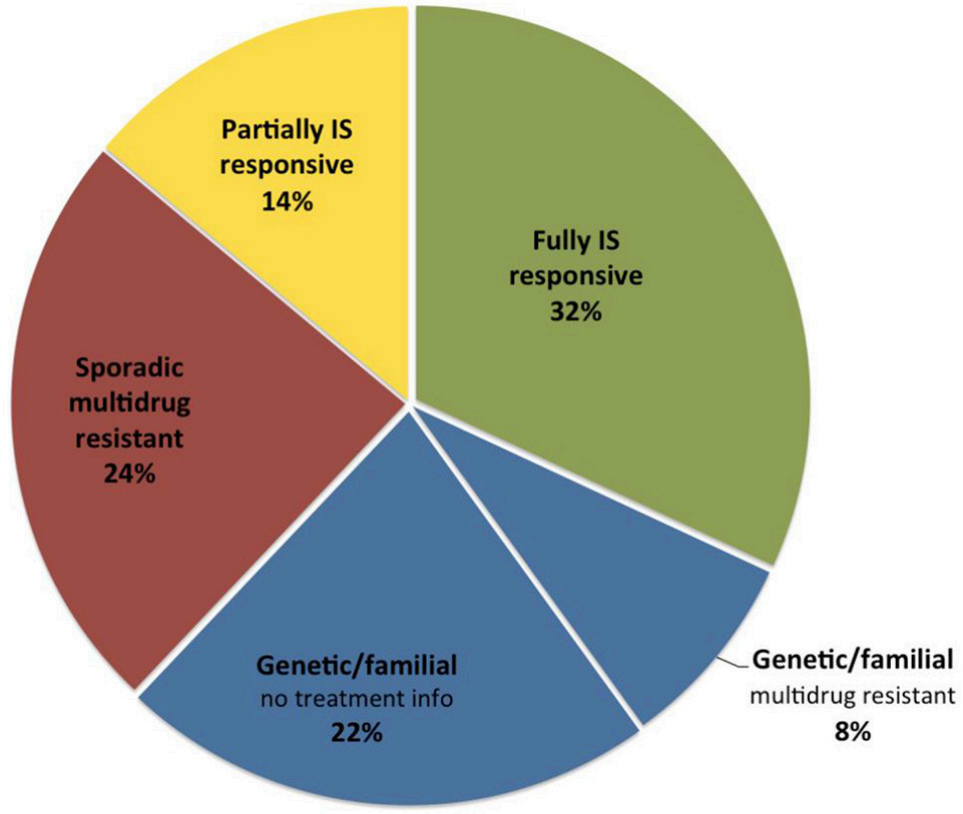
Distribution of SRNS subtypes in the PodoNet cohort according to etiology and treatment responsiveness.

Historically, the prognosis of SRNS was mainly staged according to histopathologic findings, with limited predictability of medium- and long-term disease outcomes (41–46). Providing comprehensive clinical and biochemical information with up to 15 years of follow-up, the PodoNet database allows to assess the prognostic effect of early treatment responsiveness in the context of genetic and histopathologic findings, which provide a rationale for a evidence-based reclassification of SRNS into sporadic immunosuppression-responsive (32%), sporadic multidrug-resistant (24%), genetic, and familial SRNS (30%)(Figure 7).

The average overall ESKD-free survival of patients with primary SRNS in the PodoNet cohort was 74% at 5 years, 58% at 10 years and 48% at 15 years, in keeping with previous cohort studies in which 5-year renal survival ranged from 65 to 92% and 15-year survival from 34 to 72% (43, 45, 47–49).

**Figure 8:**
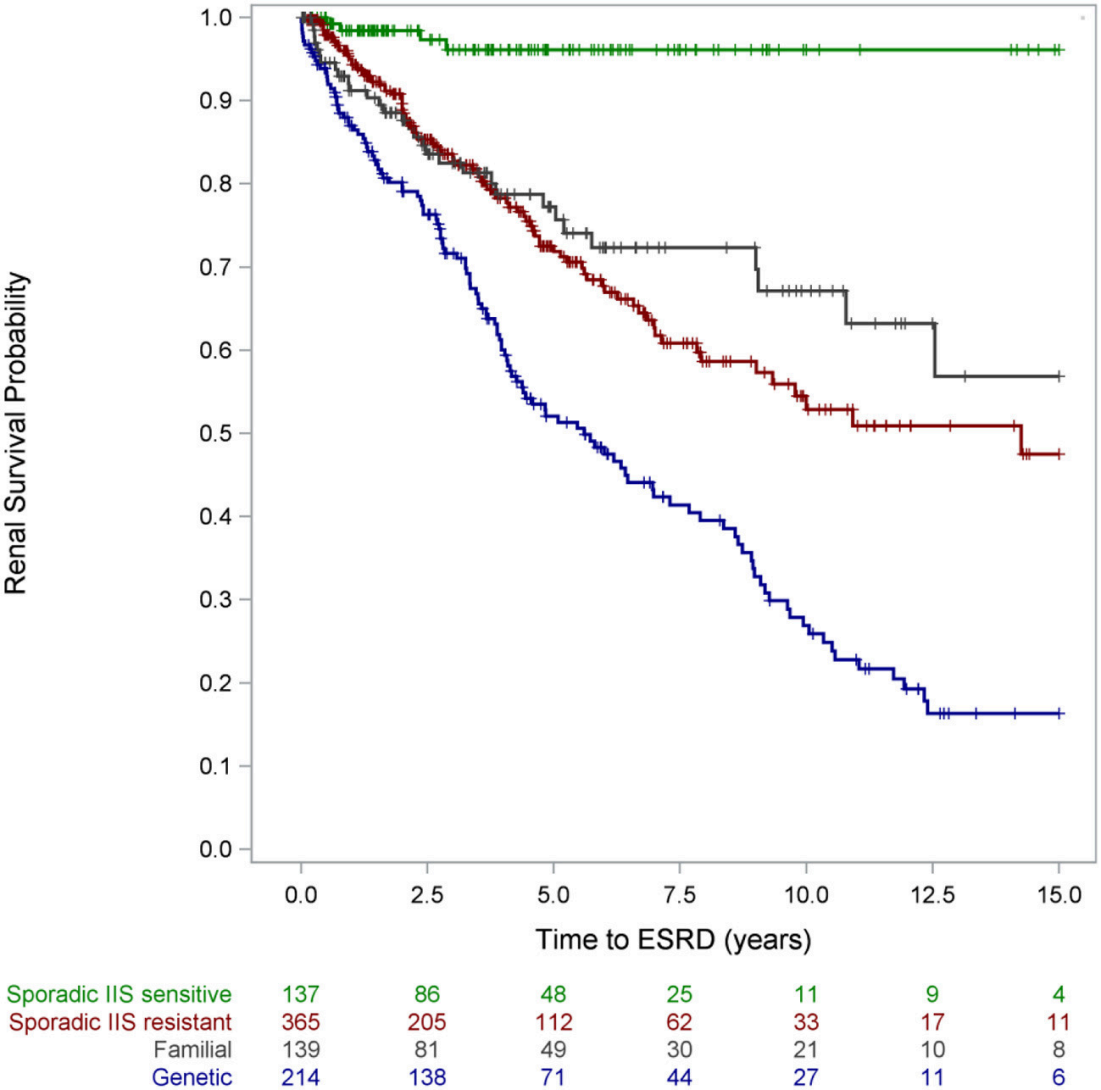
Renal survival by disease category. Patients with partial responsiveness to intensified immunosuppressive (IIS) therapy were classified IIS resistant for this analysis [with copyright permission (2)].

Responsiveness to intensified immunosuppression was highly predictive of long-term outcomes: Whereas complete remission during the first treatment year was associated with 94% 15-year renal survival, only 37% of the multidrug resistant patients were not in end-stage disease 15 years after disease onset (2). A fraction of patients achieved partial remission; it is controversial whether such intermediate response patterns reflect pharmacological effects on the immune system or non-specific proteinuria-lowering effects of RAS antagonists and possibly calcineurin inhibitors. Notably, partial proteinuria reduction was associated with superior long-term renal survival compared to patients with multidrug resistant proteinuria. The other key determinant of long-term renal outcome was the identification a genetic disease cause (2): Three quarters of patients diagnosed with a hereditary podocytopathy progressed to end-stage kidney disease within 15 years, as compared to only 4% of SRNS patients with sporadic disease and responsiveness to immunosuppressive therapy (Figure 8). Long-term renal outcomes were remarkably similar in children with different genetic disease entities. The 10-year renal survival rate was 72% for *NPHS2*-associated nephropathy, 77% for *WT1*-associated disease and 71% for the less common podocytopathies (2).

Remarkably, the outcome of children with proven genetic disease was also inferior compared to patients with multidrug-resistant disease in whom no genetic diagnosis could be established. The prognostic values of genetic diagnosis and immunosuppression responsiveness were found to be independent by multivariate analysis: ESKD risk was increased by 150% in patients in whom a genetic diagnosis was established whereas complete remission reduced renal risk by 87% in patients who achieved complete remission and by 50% in those who responded partially to immunosuppressive therapy (2).

**Figure 9:**
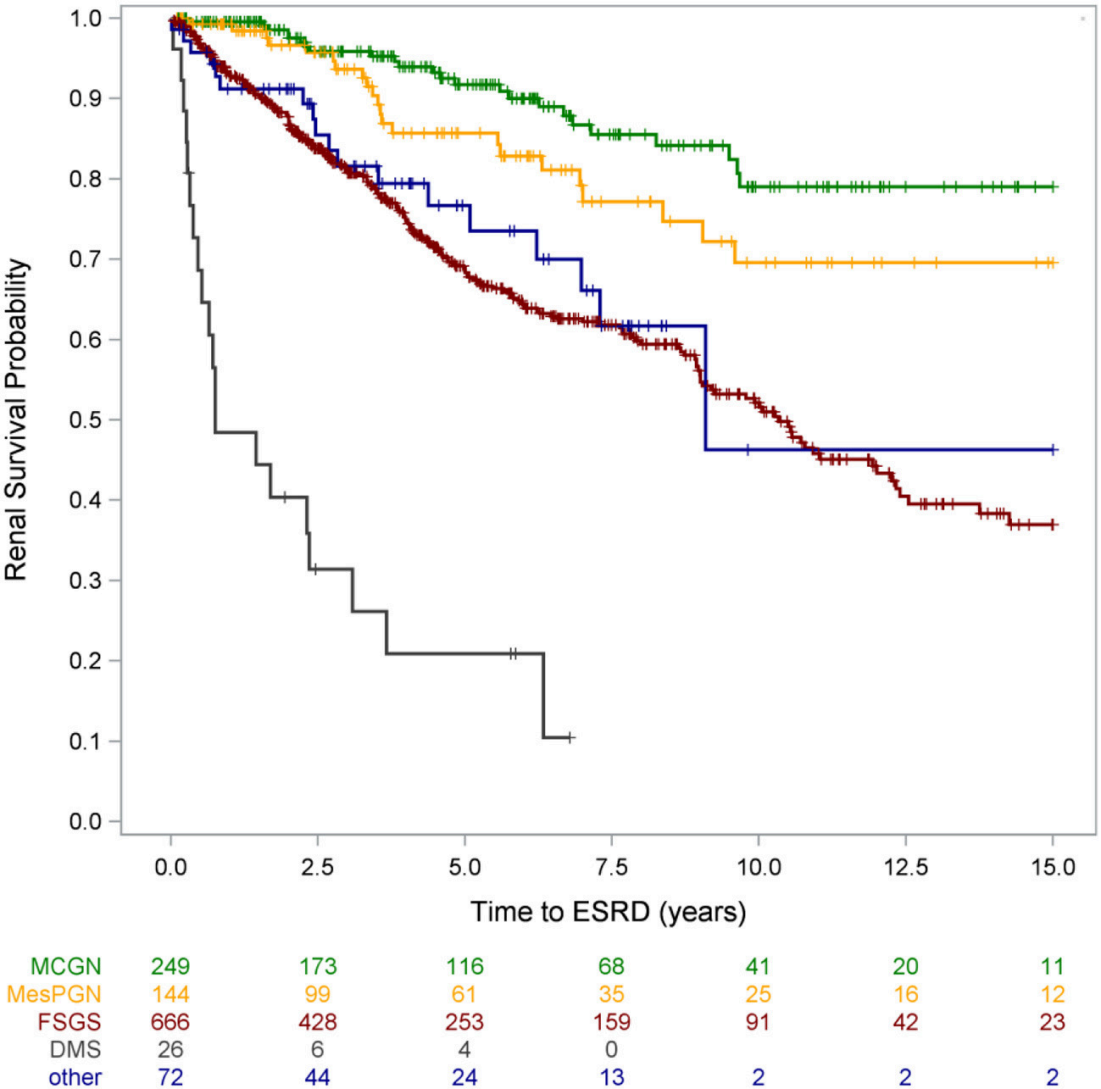
Renal survival by histopathological diagnosis [from Trautmann et al. (2) with copyright permission, supplemental materials].

Traditionally, the diagnostic categorization and prognostic evaluation in SRNS was based on histopathologic diagnosis. As expected, we found strong associations between histopathologic findings at time of diagnosis and long-term renal survival: ESKD-free survival was significantly higher for MCN than FSGS (79% v. 37% at 15 years) (Figure 9), and the overall ESKD risk of children diagnosed with FSGS was 4-fold higher compared to MCN (2). Children diagnosed with DMS showed the poorest outcomes, with a 20-fold increase of ESKD risk relative to MCN with progression to ESKD in 80% within 5 years after initial SRNS manifestation. Importantly, the prognostic value of FSGS and DMS prevailed even when adjusting for CKD stage at diagnosis, responsiveness to intensified immunosuppression, and the presence or absence of a genetic diagnosis (2). For instance, a patient with a genetic podocyte disorder, multidrug resistance, and a given eGFR will still have a nearly threefold higher ESKD risk with the diagnosis of FSGS compared with MCN. Hence, the independence of genetic and histopathological findings in the multivariate risk analysis suggests that histopathological assessment is still relevant in the genetic era.

Additional independent risk factors of progression to ESKD were identified as age <1 year and older than 5 years at disease onset and advanced CKD at initial presentation (2).

### Which lessons have we learned from the podonet registry so far?

The understanding of the underlying molecular, genetic and pathophysiological mechanisms of SRNS has profoundly advanced during the past decade. Established in 2009, the PodoNet registry has grown to one of the largest pediatric SRNS cohorts with more than 2000 children feeding clinical and genetic research. While the large size of the cohort is a major asset, the incompleteness of data reporting and biosample availability has been a limitation to data analysis and interpretation. Also, in the early years of the Network technological limitations and restricted resources hampered genetic screening. These limitations were partially overcome in later years thanks to improved funding and the central implementation of podocyte gene panel sequencing.

The discovery of numerous novel genetic podocytopathies and the progress in diagnostic screening technology have already changed clinical practice during the time span of the PodoNet project. Initially, children underwent a diagnostic algorithm and were selectively screened for individual SRNS genes by means of Sanger sequencing based on clinical criteria and depending on age at disease onset. With the advent of NGS technologies genetic screening has become much more rapid, comprehensive and cost-effective and consequently rapidly transgressed from an optional adjunct to a key component of the diagnostic workup in SRNS. This transformation is well-reflected in the output of PodoNet as well as other study consortia (10).

The PodoNet network has been an active player in this field by contributing to gene discoveries, developing and systematically applying podocytopathy gene panels, and analyzing phenotypes and outcomes by comprehensive assessment of clinical, genetic, and histopathological findings as well as responsiveness to pharmacotherapies.

**Figure 10:**
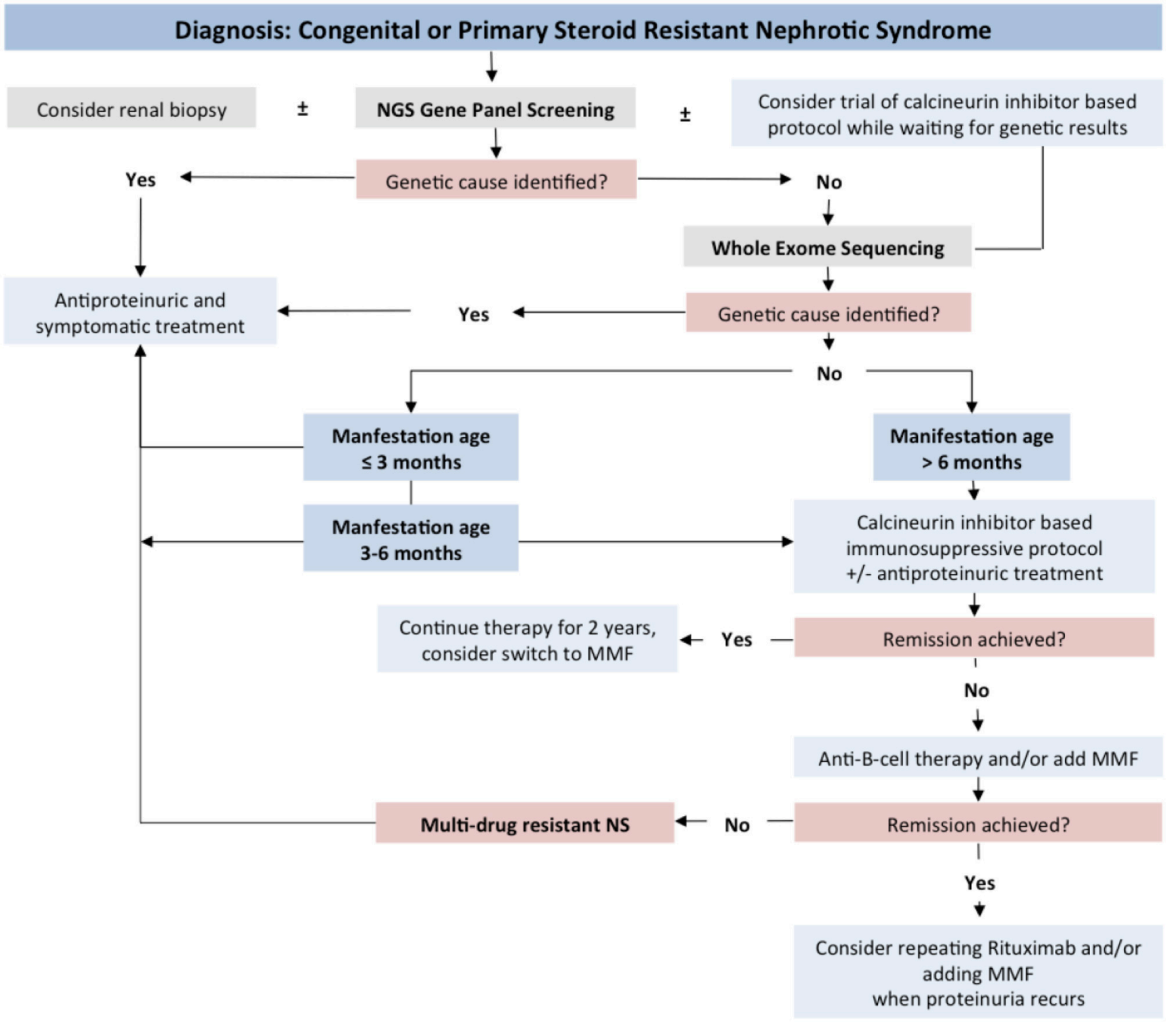
Proposed diagnostic and therapeutic algorithm for children with CNS/SRNS.

Our studies allow concluding that genetic screening should be initiated as soon as the diagnosis of steroid resistance has been made, in parallel to renal biopsy (Figure 10).

We obtained evidence that genetic forms of SRNS are largely non-responsive to intensified immunosuppressive therapy, which, in consequence, should be avoided in patients with genetic disease. Future research will address in detail the short- and long-term usefulness of RAS inhibition and other supportive treatment strategies in genetic podocytopathies.

Our studies also have highlighted the lacking specificity of the most common histopathological diagnoses with respect to identifying underlying etiologies. On the other hand, the histopathological diagnosis was found to retain some prognostic value even when the genetic status of a patient is known.

In immune-mediated forms of SRNS, calcineurin inhibition was demonstrated to be the most efficacious second-line therapy following the diagnosis of steroid resistance. Children resistant to calcineurin inhibitors are usually also resistant to other immunosuppressive agents. Moreover, initial responsiveness to calcineurin inhibitors is uniquely predictive of long-term preservation of kidney function.

The insights obtained from the PodoNet cohort will facilitate the development of rational evidence based clinical practice recommendations for SRNS. In view of the major prognostic differences associated with genetic findings and pharmacotherapeutic responsiveness, we propose to sub-classify SRNS patients as sporadic immunosuppression-responsive, sporadic multidrug-resistant, genetic, and familial SRNS.

## Author contributions

AT, BL-Z, FS contributed substantially to the acquisition, analysis, and/or interpretation of the data, participated actively in preparing the manuscript and approved the submitted final version.

### PodoNet collaborators

**Austria:** Dagmar Csaicsich; Chile: Marta Azocar, Santiago; Lily Quiroz, Santiago; **Colombia**: Lina Maria Serna Higuita, Medellín; **Czech Republic**: Jirí Dušek, Prague; **France**: Bruno Ranchin, Lyon; Michel Fischbach, Strasbourg; **Georgia**: Tinatin Davitaia, Tbilisi; **Germany**: Jutta Gellermann, Berlin; Sandra Habbig, Cologne; Jun Oh, Markus J. Kemper, Hamburg; Anette Melk, Hannover; Agnes Trautmann, Franz Schaefer, Heidelberg; Hagen Staude, Rostock; **Greece:** Nikoleta Printza, Thessaloniki; **Hungary**: Peter Sallay, Budapest; **Iran:** Alaleh Gheissari, Isfahan; **Italy**: Marina Noris, Bergamo; Andrea Pasini, Bologna; Gian Marco Ghiggeri, Monica Bodria, Genova; Gianluigi Ardissino, Milano; Elisa Benetti, Padova; Francesco Emma, Rome; **Lebanon**: Bilal Aoun, Beirut; Pauline Abou-Jaoudé, Byblos; **Lithuania**: Augustina Jankauskiene, Vilnius; **Poland**: Anna Wasilewska, Bialystok; Ewa Gacka, Chorzow; Beata S. Lipska-Ziętkiewicz, Aleksandra Zurowska, Gdansk; Dorota Drozdz, Krakow; Marcin Tkaczyk, Małgorzata Stanczyk, Lodz; Halina Borzecka, Lublin; Magdalena Silska, Poznan; Tomasz Jarmolinski, Szczecin; Agnieszka Firszt-Adamczyk, Torun; Mieczyslaw Litwin, Elzbieta Kuzma-Mroczkowska, Hanna Szymanik-Grzelak, Warsaw; Anna Medynska, Wroclaw; Maria Szczepanska, Zabrze; **Portugal**: Alberto Caldas Afonso, Porto; Helena Jardim, Porto; **Romania**: Adrian Lungu, Bucharest; **Serbia:** Amira Peco-Antic Belgrade; Radovan Bogdanovic, Belgrade; **Sweden:** Rafael T. Krmar, Stockholm; **Switzerland**: Sybille Tschumi, Bern; **Syria:** Bassam Saeed, Damascus; **Turkey:** Ali Anarat, Adana; Ayse Balat, Gaziantep; Z. Esra Baskin, Ankara; Nilgun Cakar, Ankara; Ozlem Erdogan, Ankara; Birsin Özcakar, Ankara; Fatih Ozaltin, Ankara; Onur Sakallioglu, Ankara; Oguz Soylemezoglu, Ankara; Sema Akman, Antalya; Faysal Gok, Gulhane; Salim Caliskan, Istanbul; Cengiz Candan, Istanbul; Alev Yilmaz, Istanbul; Sevgi Mir, Izmir; Ipek Akil, Pelin Ertan, Manisa; Ozan Özkaya, Samsun; Mukaddes Kalyoncu, Trabzon; **United Arab Emirates:** Eva Simkova, Loai Akram Eid, Dubai; **Ukraine:** Svetlana Fomina, Kiev; Roman Sobko, Lviv.

### Conflict of interest statement

The authors declare that the research was conducted in the absence of any commercial or financial relationships that could be construed as a potential conflict of interest.
